# Chronic hepatitis associated with antiribosomal‐P autoantibodies in a 14‐year‐old girl

**DOI:** 10.1002/ccr3.2410

**Published:** 2019-09-05

**Authors:** Coline Mejean, Daniel Bertin, Kim Guetta, Cécile Desaldeleer, Mathilde Butori, Bertrand Roquelaure, Alexandre Fabre

**Affiliations:** ^1^ Service de pédiatrie multidisciplinaire Hôpital de la Timone enfants, AP‐HM Marseille France; ^2^ Service d’immunologie Hôpital de la Conception, AP‐HM Marseille France; ^3^ Service d’anatomopathologie Hôpital de la Timone enfants, AP‐HM Marseille France; ^4^ Unité d’hépato‐gastro‐entérologie pédiatrique Fondation Lenval Nice France; ^5^ Aix Marseille Univ, INSERM, MMG Marseille France

**Keywords:** antiribosomal‐P antibodies, anti‐RibP, autoimmune hepatitis, children, lupus hepatitis

## Abstract

We reported the first pediatric case of auto‐immune hepatitis with positive anti‐P antibodies. On the basis of our findings, adding auto anti‐P screening in pediatric seronegative HAI may be recommended.

## 1 DESCRIPTION OF IMAGES

A 14‐year‐old girl presented with chronic cytolytic hepatitis with icterus and asthenia. Ultrasonography revealed hepatomegaly without cirrhosis or portal hypertension. The results of blood tests, metabolic investigations, blood copper analysis, and toxicology analysis were normal. Serum autoantibodies of autoimmune hepatitis (AIH) were not detected. However, hypergammaglobulinemia (20 g/L) was present. Thus, AIH was still suspected.

**Figure 1:**
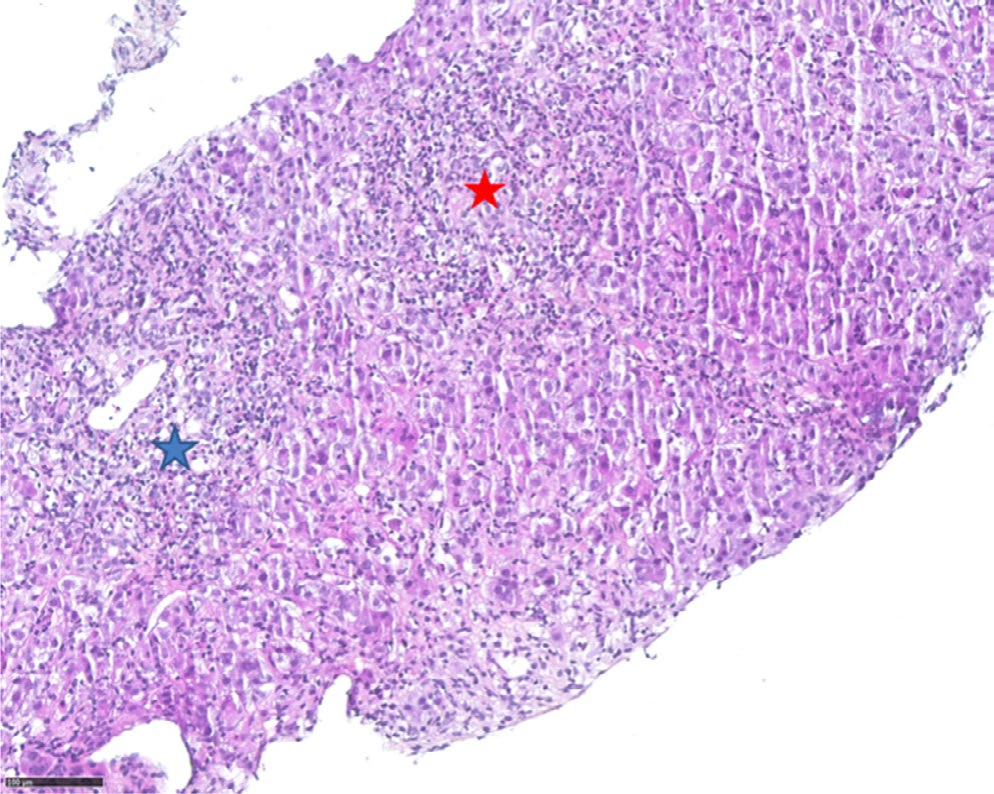
Histology with hematoxylin‐eosin‐saffron staining. Polymorphic inflammatory infiltration (chronic lymphocytic and acute population with neutrophil and polynuclear cells). Disposition of peri‐centrilobular (blue star) and lobular (red star) areas of the liver, which is not typical of autoimmune hepatitis. Scale bar: 100 µm

**Figure 2:**
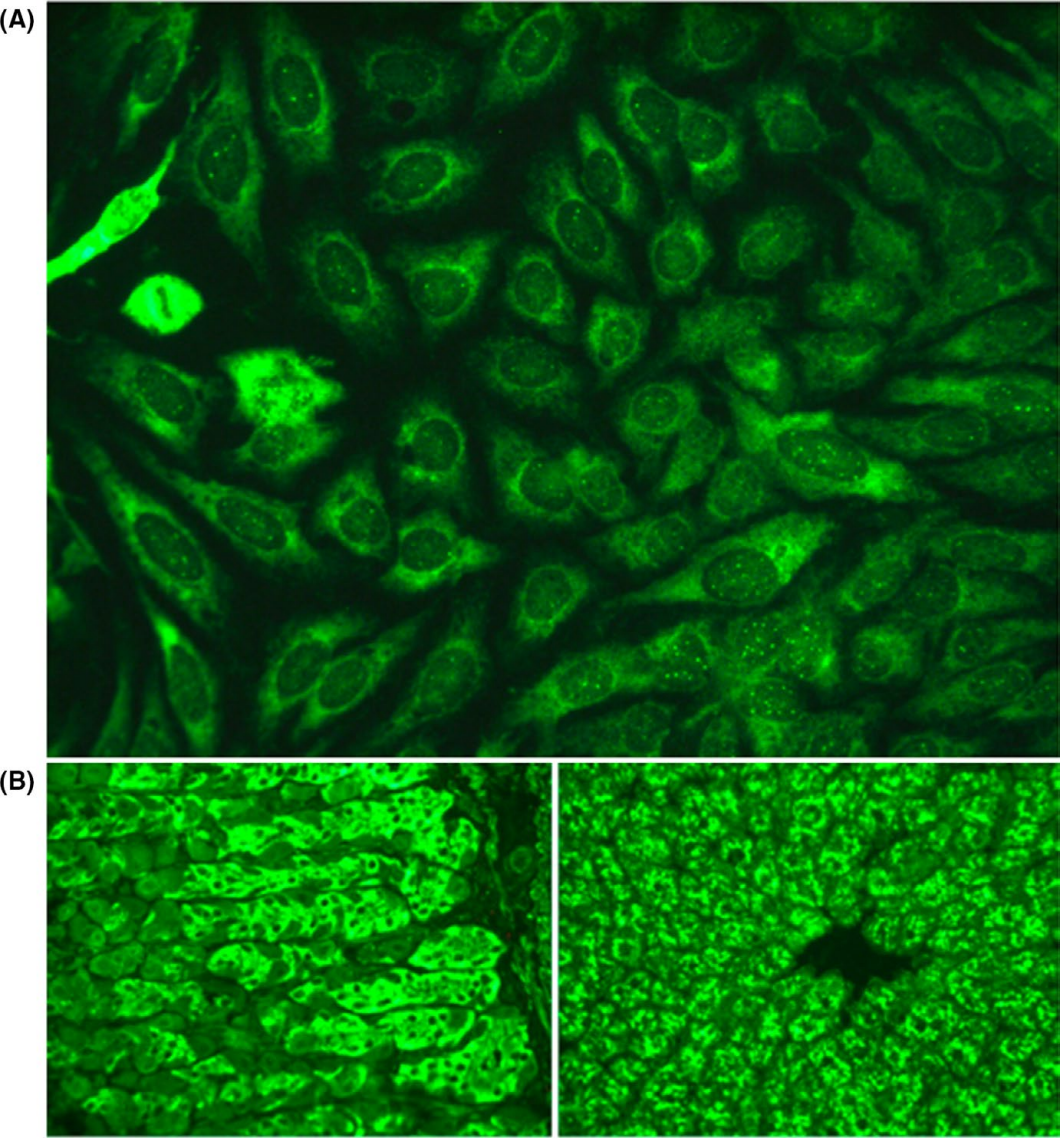
A, Indirect immunofluorescence (IIF) pattern observed on HEp‐2 cells (original magnification × 400): Fluorescent cytoplasmic staining of HEp‐2 cells compatible with antiribosomal‐P autoantibodies. Antinuclear autoantibodies are also detectable through fluorescent discrete speckles visible in the nucleoplasm (nuclear dot pattern). B, Typical antiribosomal‐P pattern on rat liver‐kidney‐stomach substrate by IIF (original magnification × 400). (Left): Stomach substrate showing fluorescence in the cytoplasm of chief cells of the gastric mucosa. (Right): Liver substrate showing cytoplasmic clumped fluorescence around hepatocyte nuclei

Liver biopsy revealed an inflammatory infiltrate (Figure 1) with fibrosis, without evidence of primary biliary cirrhosis. Biological screening for autoantibodies revealed a fluorescence pattern compatible with antiribosomal‐P autoantibodies (Figure 2). Fluorescence enzyme immunoassay confirmed the presence of antiribosomal‐P autoantibodies, which are mainly known to be lupus‐specific^1^ and have shown little association with autoimmune hepatitis. Only one study has reported the presence of these autoantibodies in 10% of adults with AIH.^2^ From diagnosis until 1‐year follow‐up, no other clinical manifestations of lupus occurred.

Budesonide and azathioprine were administered in this patient. The patient's transaminase levels normalized after 2 months.

To conclude, this could be the first reported case of pediatric AIH with negative common laboratory investigation results but positive antiribosomal‐P autoantibodies. On the basis of our findings, adding antiribosomal‐P autoantibodies to the screening panel for AIH may be recommended.

## CONFLICT OF INTEREST

None declared.

## AUTHOR CONTRIBUTIONS

CM: served as primary author, making sure that the data are accurate, that all deserving authors have been credited; and responsible for the literature review and final approval, submitting revisions and final version, and communicating with editors. DB: served as secondary author, was responsible for the immunofluorescence images and made final edits before the submission. KG, coauthor: was responsible for histologic images. BR:was responsible for data collection and analysis, manuscript drafting, and literature review. CD and MB: critically edited and revised the initial draft of the manuscript with regard to important intellectual content. AF: was responsible for supervision, guidance, and final approval of the manuscript. All authors discussed the case and commented on the manuscript at all stages and gave their final approval of the version to be published in Clinical Case Reports.

## CONSENT

The patient has provided written consent for the case report to be published.
